# The Book World of Medicine and Science

**Published:** 1900-09-15

**Authors:** 


### The Book World of Medicine and Science.

The Early Treatment of Appendicitis. By Donald W. C. Hood, M.D., F.R.C.P., Senior Physician, West London Hospital. 8vo., 38 pp. (London: John Bale, Sons, and Danielsson, Ltd. Price 2s. 6d. net.)

This little book consists of a lecture delivered at the West London Post-Graduate College in 1897, revised and partially rewritten. It is a plea for the early and medical treatment of appendicitis. The author's text, as it were, is, given early diagnosis, then the consistent and thorough methods of the physician will not only prevent the onset of serious complications, but in the larger proportion of cases lead to perfect recovery. While fully admitting the value of operative measures in many of the conditions resulting from appendicitis, yet he considers that the apparently increasing death-rate of the disease is to be explained by improper treatment at its inception. In discussing the diagnosis in the early stages, he relies upon pain, some elevation of temperature, absence of movement of the abdominal wall over the affected area, localised tenderness, and the formation of a definite swelling. There is nothing, he believes, that will indicate at the commencement of an attack whether it is going to terminate in resolution or suppuration, and it is perhaps in this admission that the strength of his subsequent argument somewhat dwindles. The author deplores the difference of opinion which exists at the present day as to the appropriate treatment of appendicitis in its primary stages, but seeing that there is this want of harmony, he feels that there is no apology needed for holding a strong personal conviction as to the most judicious method of dealing with the affection. Half measures will not do, for by their use the patient may be lost. He pins his faith to opium, slop diet, and absolute rest, but these means must be employed at the incipient period if they are to be successful.

Modern Asylum Construction. By R. H. Steen, M.D. (Adlard and Son. 8vo., pp. 16.)

In this little pamphlet Dr. Steen follows up the important subject of asylum construction, and he is severe, but not too severe, in his remarks on two of the three methods of procedure adopted when a new asylum is about to be built. Whether the third method is always successful is doubtful, unless the medical expert has been associated with the architect in the preparation of the plans from the very beginning. "Watchful care" as construction is proceeding would probably be insufficient, and would certainly be expensive. Dr. Steen divides asylum construction into two systems, the "connected" and the "detached," and he sums up in favour of the former, modified perhaps, but still the "connected" system, and we agree with him entirely. The hope of seeing a really first-class county asylum in this generation lies almost dead within us; and we think Dr. Steen should have stated his views on the modern system of selection of the plan which is to be carried out, which is often anything but satisfactory.

Experiments on Animals. By Stephen Paget, with an Introduction by Lord Lister. (London: T. Fisher Unwin. 1900. Price 6s.)

This is not essentially a medical book. It is written for general readers in order that they may understand and carry in their minds the facts of the case in regard to the subject with which it deals; a matter of no small importance in view of the extraordinary absence of fact which characterises most of the discussions on vivisection which, with weary reiteration, crop up again and again in some of our daily contemporaries. The book before us has the great advantage of not being written by a man who is himself a "vivisector," but by one who, from the exceptional opportunities which have fallen in his way of judging of the importance of the investigations which depend for their very being upon the power of bringing things to the final test of experiment upon living animals, has, as Lord Lister says in his introduction, been deeply impressed with the greatness of the benefits which these experiments, and the investigations of which they form an essential part, have conferred upon mankind, and with the grievous mistake which is being made by those well-meaning, perhaps, but misguided people who desire to suppress all such methods of inquiry. The readers of this book will see not only how exaggerated is the view that some people take of the nature of some of the experiments themselves—although this is not a point on which we lay much stress—but also how great has been the benefit which has resulted from their performance. The book is written in an interesting and graphic style, it is full of facts, and it will serve well to give to the general reader, and moreover to all who wish to know before they talk, a good and common-sense view of the whole subject.

Things Seen: Impressions of Men, Cities, and Books. By G. W. Steevens. Selected and Edited by G. S. Street, with a Memoir by W. E. Henley. (Edinburgh and London: William Blackwood and Sons. 1900. Price 6s.)

Among the losses which the nation has suffered at the hand of war, a loss felt almost as a personal one by thousands of readers, has been the death of G. W. Steevens. It was in newspaper form that most of us were familiar with his writings, but none can doubt that if he had been spared a brilliant future and many triumphs probably not confined to literature would have fallen to his lot. This book consists of articles from newspapers and magazines, and deals with a wide variety of subjects, each of which serves to show the still wider variety of knowledge with which the writer's mind was stored. The war in Thessaly, Zola, the Dreyfus case, the Jubilee, the Bayreuth festival, the Irish famine, prisons, hospitals, races, all come alike to this wonderful pen, and on all alike the author has something to say which is virile and striking, and which serves to impress upon one not only the facts described, but the lessons which they teach. Medical students who would see what it is possible for a careful observer, all awake, to see and pick up from a single visit to a hospital should read the last chapter of the volume, describing as it does a morning in an out-patient department and an afternoon in an operating theatre. Would that the average student had such eyes and such an understanding. One cannot but feel that what he had once seen he must have been able to visualise afresh, and that while writing the whole thing must have stood again like a picture before him. Surely a faculty to cultivate.

Golden Rules of Ophthalmic Practice. By Gustavus Hartridge, F.R.C.S. Golden Rules Series No. VII. (Bristol: John Wright and Co. Price 1s.)

Yet another of the "Golden Rules" series of little manuals, and this time a very good one. As the author very properly says, disastrous mistakes are more easily made in connection with diseases of the eye than in any other branch of medical science, especially by those in general practice whose opportunities of seeing eye cases are few and far between. In this little book no attempt is made to cover the whole field of ophthalmology, reference being chiefly made to the important points on which the student is most apt to go astray, and with that limitation always before us we can only say that the work is very well done, and that the booklet is likely to be found useful by those for whom it is intended.

